# Daily Intake of *Lemna minor* or Spinach as Vegetable Does Not Show Significant Difference on Health Parameters and Taste Preference

**DOI:** 10.1007/s11130-022-00952-9

**Published:** 2022-02-12

**Authors:** Jurriaan J. Mes, Diederik Esser, Dianne Somhorst, Els Oosterink, Sandra van der Haar, Meeke Ummels, Els Siebelink, Ingrid M. van der Meer

**Affiliations:** 1 Wageningen Food & Biobased Research, Wageningen University & Research, Bornse Weilanden 9, 6708 WG Wageningen, The Netherlands; 2 Division of Human Nutrition & Health, Wageningen University & Research, Stippeneng 4, 6708 WE Wageningen, The Netherlands; 3 Wageningen Plant Research, Bioscience, Wageningen University & Research, Droevendaalsesteeg 1, 6708 PB Wageningen, The Netherlands

**Keywords:** Duckweed, Sustainable vegetable, Safety, Tolerance, Adverse events, Taste

## Abstract

The growing world population will increase the demand for new sustainable foods and ingredients. Here we studied the safety and tolerance of *Lemna minor,* a new sustainable vegetable crop from the duckweed family. Twenty-four healthy adults consumed either *L. minor* plant material or spinach as vegetable (170 g fresh weight) as part of a warm meal on 11 consecutively days in a randomized controlled parallel trial design. The intervention meals had a different recipe for each day of the week. All participants had to report daily if they experienced gastric complaints, feelings of hunger, fullness, desire to eat, thirst, general health, nausea, and stool consistency. Only hunger, flatulence and constipation were significantly different between both intervention groups. At the start and end of the intervention, blood and urine were sampled in order to analyze biomarkers for general health, *e.g*., kidney function, liver function, cardiovascular health, inflammation and iron status. Both intervention groups did not show significant differences for these biomarkers. In taste attributes the *L. minor*-based products showed in only a few specific cases a significant difference compared to the spinach-based products. Based on the results we conclude that 11 consecutive days intake of 170 g fresh weight *L. minor* plants as a cooked vegetable does not result in any adverse effect in healthy adult subjects.

**Supplementary Information:**

The online version contains supplementary material available at 10.1007/s11130-022-00952-9.

## Introduction

The growing world population will demand a larger and more efficient food production [1]. Food that can be produced with low foot print and resilience against climate variation, that could be produced in urban areas or by vertical farming, and that can provide high nutritional value with a high protein content, will become of growing importance. Duckweed is a plant family that might fit all of these requirements. Firstly, duckweed has a very high vegetative growth rate, it can grow almost everywhere in the world and can tolerate extreme environmental circumstances [2–6]. Secondly, it can be cultivated outdoors in a basin, in greenhouses or in multi-layer vertical farming systems, thereby not making use of agricultural farming land [6]. Thirdly, duckweed contains high amounts of protein (35–43% on dry weight basis), with a profile of essential amino acids that matches the requirements for humans better than most vegetable crops [5–7]. Protein concentrate of duckweed also has the potential to be well digestible [8]. Fourth, it is rich in micro and macro elements, vitamins, carotenoids and specific flavonoids and has a favorable fatty acid composition [9, 10]. Therefore duckweed can be considered a sustainable and nutritious food source.

Duckweed has been used for a long time as animal feed [3, 10, 11] without any adverse effects. Besides that, duckweed has been consumed by humans for generations as a nutritious vegetable (named ‘Khai-Nam’) in Southeast Asian countries [12]. The duckweed family can be divided into five genera (Spirodela, Landoltia, Lemna, Wolffia and Wolffiella) and 38 species [2]. According to the literature, *Wolffia arrhiza* and *Wolffia globosa* are consumed most in Southeast Asian countries but *L.minor, L. gibba,* Landoltia and Spirodela with their larger leaves might have a high potential as new sustainable vegetable crop.

In a previous study, we found that humans can absorb less proteins from *L. minor* plant material than from green pea [13]. Kaplan et al. [14] concluded that *W. globosa* had an almost similar bioavailability compared to a green pea-based product. Importantly, no adverse effects or intolerance were reported by the volunteers during controlled trials with single intake [13–15]. These *in vivo* experiments were supported by *in vitro* analysis on toxicity demonstrating no detectable anti-proliferative or cytotoxic effects [16]. As all genera of duckweed are classified as Novel Food by the European Food and Safety Authority, it is needed to gain more certainty on the safety of this novel food. Developing a new sustainable food requires a sustainable method to investigate safety, preferably without using animal studies. Human trials are a more appropriate method to analyze products with an expected very low risk for the volunteers.

The primary objective of this study was to analyze tolerance of healthy adult subjects towards an intake of a novel food compared to an established food product with similar food application characteristics. Other than 90 days animal toxicological studies, no consensus *in vivo* protocols are yet available to analyze the safety aspects for novel food products. We studied the safety of food products based on a realistic intervention, with food products consumed regularly but with an intake higher than one will implement in a normal and variable diet. Human trials also allow to include sensory information which is a crucial factor to success. Therefore, a human trial was performed in order to analyze the effect on general health parameters but also on taste attributes during 11 consecutively days of intake of *L. minor* as vegetable.

## Materials and Methods

### Subjects

A total of 24 healthy subjects were recruited via the database of Wageningen Food & Biobased Research. Inclusion criteria were: age between 18 and 50 years, BMI between 19 and 25 kg/m^2^ and apparently healthy as assessed by a health and lifestyle questionnaire. Subjects were excluded in case of any metabolic, gastrointestinal, inflammatory or chronic disease, a history of gastro-intestinal surgery, liver dysfunction, liver surgery or kidney dysfunction. Other exclusion criteria were: use of gastric acid inhibitors or laxatives, use of hard drugs, taking more than ≥4 glasses of alcoholic beverages per day (in average), being pregnant or lactating, and being a current smoker. Also subjects who had strong food allergies or who were on a slimming, medically prescribed or vegan/ vegetarian diet were excluded.

### Study Design

The study was a randomized controlled parallel trial, in which study subjects daily consumed either *L. minor-* or spinach-based meals for a period of 11 consecutive days. Spinach was chosen as a control intervention since this known vegetable is well appreciated by consumers and has similar properties for meal applications. Warm meals were consumed for lunch and were offered in different forms throughout the trial. Meals were provided in the following order: pasta, curry, mashed potato, risotto, soup, lasagna, quiche, pasta, curry, mashed potato, risotto, which was equal for both groups. Each meal contained either 170 g fresh weight (FW) *L. minor* or 170 g spinach. Meals were consumed at the Health Research Unit during week days and ready-to-eat meals were provided for weekend days. Before and after the intervention a fasting blood sample and a morning urine sample were collected and blood pressure was measured. Study subjects were instructed to maintain their habitual diet and activities during the intervention. Subjects were stratified among groups based on gender, age and BMI resulting in no statistical differences between the two groups for these parameters (supplementary Table S1). All subjects completed the intervention study.

### Products

*Lemna minor* (accession 8623) was produced under strict hygiene conditions as reported previously [13] but tap water was replaced by demineralized water in order to prevent copper and arsenic accumulation in plant material. *L****.*** *minor* plants were grown in 10 L trays in an indoor growth chamber on a defined stagnant medium and light regime as previously reported [13] but without UV filter, aeration and water circulation. Harvested plant material was washed and frozen at -20C° until further use. The plant material was analyzed for nutritional, microbiological and toxicological content (Nutrilab Giessen; Wageningen Research). A general overview of the nutrient composition of *L. minor* is provided in supplementary Table S2. Based on these results, no harm was expected for frequent intake by the study subjects. The spinach was commercially available frozen spinach (Bonduelle, the Netherlands) and nutritional data were taken from the Dutch food composition database (NEVO).

Both frozen products, *L. minor* and spinach, were heated in a steam oven (100 °C) for 20 min and per subject 170 g was added to the basic meal ingredients. Other ingredients of the meals can be found in supplementary Table S3. The energy content of the meals was between 450 and 500 kcal. In addition to the meal provided, the participants were completely free in their choice of food.

### Procedures

Participants were requested to complete a questionnaire on health and eating behavior at the start of the intervention. Participants visited the research facility (study day 1) under fasting conditions, heart rate and blood pressure were measured with a blood pressure monitor (Omron HEM 907). A urine sample and blood sample was collected. Then subjects were offered a light breakfast and they completed a food frequency questionnaire. Later that day, participants received their first intervention meal. The next consecutive ten days subjects consumed the intervention meals and completed the questionnaires. Study subjects were not allowed to talk to each other during these lunches. Subjects completed another online questionnaire on satiety and gastro-intestinal complaints at the end of the afternoon, before the evening meal. On study day 12, subjects came to the facility again in the morning in fasted state and again urine and blood samples were collected and blood pressure and heart rate were measured. On day 12, 13 and 14 subjects also completed a questionnaire on gastro-intestinal complaints as during the previous days.

### Measurements

Primary outcomes were gastro-intestinal complaints during the intervention period scored on a 10 cm visual analogue scale (VAS) representing values from 0 to 100, with the anchors from ‘not at all present’ to ‘strongly present’, respectively. The following parameters were reported: hunger, fullness, desire to eat, can eat, thirst, bloating, belching, abdominal pain, flatulence, nausea, diarrhea and constipation. All other adverse events (AEs) were registered by self-reporting and questioning by a medical doctor (MD). AEs were classified under the responsibility of the MD according to ICD-10 coding.

Blood Hb, glucose, Fe, leukocyte cell counts, ALAT and GGT (liver), CRP, zonulin (gut integrity markers) were analyzed at the hospital the Gelderse Vallei in Ede, using standardized clinical procedures. Creatinine and eGFR (kidney) were analyzed in spot urine samples. Systolic blood pressure (sBp), diastolic blood pressure (dBp) and heart rate (HR) were assessed automatically (DINAMAP® PRO 100) for 10 min with a 3 min interval after a 10 min rest.

### Analysis of Sensory Characteristics

Evaluation of sensory attributes (pleasant aroma, overall liking, good taste, good mouth feel nice after taste) and willing to try again was performed each by a 10 point scale.

### Statistical Analysis

Statistical analyses were performed using SPSS statistics. In general, data is presented as mean ± standard deviation (SD). Daily changes in complaints during the intervention period were compared between interventions by using a repeated measures analysis of variance (MIXED model ANOVA). Time and intervention were used as single factors to assess main effects on traits, as well as an interaction effect time * intervention. Differences within each intervention in blood- and urine-based biomarkers and blood pressure were calculated by a paired *t*-test. To determine differences between interventions we performed an unpaired *t*-test on obtained delta values (after-before). A *p* value of *p* < 0.05 was considered as statistically significant.

## Results and Discussion

### Gastro-Intestinal Symptoms

Based on the questionnaires, a statistically significant lower hunger and higher flatulence and higher constipation was observed for the *L. minor* group (Table1). This effect partly remained during the wash out period. As the same questionnaire was not performed before the trial or after a longer wash out period we cannot rule out that these effects are fully related to the intervention or that the *L. minor* group already had a small bias towards a higher flatulence or constipation. All other parameters that were evaluated by the questionnaire were not significantly different between the two intervention groups (Table 1). Our previous study, in which the protein bioavailability of Lemna plant material was studied based on postprandial amino acids, indicated that humans could not fully utilize the available proteins from a single large bolus of 450 g boiled *L. minor* [13]. Likely this is due to the firm cell wall that is hard to crack by the human digestive system. This could reduce feeling of hunger. Besides, plant material will then enter the colon where it can be fermented by the microbiota, resulting in gas production. One might be able to adapt to the intake of *L. minor* and, like with increasing fiber intake, might benefit from a ‘run-in’ period in which the concentration is gradually increased [17]. In analogy with increasing the intake of fibers, the advice to drink more water to prevent constipation might also be relevant for high intake of *L. minor*. Although it will be very unlikely that people will consume *L. minor* plant material for 11 consecutive days in a Western world. As the trial was not blinded and subjects could noticed that they consumed the unknown *L. minor* product, these subjects might have been a bit more alert to potential gastric symptoms than those who received the spinach-based products. In the future, a cross over design would be more favorable, or participant information could be adapted and announce that both products are novel food products.

**Table 1:** Changes in satiation and GI complaints during the *L.minor* (Lm) and spinach (Sp) intervention

		Day														P- value		
Parameter	Inter vention	1	2	3	4	5	6	7	8	9	10	11	12	13	14	I	T	I*T
Hunger	L.m	50 ± 25	48 ± 28	49 ± 17	48 ± 24	68 ± 26	49 ± 29	41 ± 27	53 ± 30	49 ± 25	46 ± 29	55 ± 23	53 ± 27	52 ± 20	47 ± 27	0.01	0.12	0.73
	Sp	49 ± 23	59 ± 27	63 ± 19	63 ± 31	72 ± 15	62 ± 19	64 ± 15	64 ± 24	60 ± 21	61 ± 22	60 ± 19	64 ± 17	51 ± 26	63 ± 16			
Fullness	L.m	41 ± 20	32 ± 17	36 ± 18	40 ± 19	23 ± 16	44 ± 23	41 ± 18	36 ± 20	37 ± 18	46 ± 22	44 ± 20	34 ± 19	43 ± 20	43 ± 21	0.62	0.16	0.20
	Sp	42 ± 21	32 ± 23	39 ± 27	30 ± 21	43 ± 27	44 ± 30	36 ± 23	29 ± 18	47 ± 18	40 ± 25	29 ± 19	30 ± 19	44 ± 25	33 ± 22			
Desire to eat	L.m	62 ± 26	63 ± 23	59 ± 19	63 ± 18	76 ± 28	58 ± 27	57 ± 22	67 ± 18	63 ± 13	53 ± 24	61 ± 20	65 ± 21	58 ± 22	61 ± 23	0.09	0.53	0.65
	Sp	62 ± 15	65 ± 24	69 ± 15	76 ± 15	71 ± 19	68 ± 21	67 ± 16	70 ± 13	66 ± 14	70 ± 16	68 ± 17	66 ± 14	62 ± 20	60 ± 24			
Could eat	L.m	53 ± 23	60 ± 19	57 ± 21	56 ± 19	71 ± 21	57 ± 23	58 ± 22	56 ± 21	56 ± 20	58 ± 21	59 ± 18	62 ± 20	58 ± 19	59 ± 21	0.09	0.79	0.37
	Sp	65 ± 19	63 ± 22	63 ± 15	69 ± 19	62 ± 22	67 ± 17	62 ± 10	67 ± 13	64 ± 10	68 ± 13	62 ± 17	68 ± 12	54 ± 22	61 ± 19			
Thirst	L.m	37 ± 28	39 ± 27	45 ± 25	38 ± 30	52 ± 24	44 ± 24	36 ± 27	39 ± 34	45 ± 23	31 ± 28	39 ± 32	42 ± 23	52 ± 24	48 ± 26	0.84	0.62	0.24
	Sp	43 ± 22	48 ± 24	41 ± 23	47 ± 23	47 ± 22	49 ± 29	38 ± 26	40 ± 26	34 ± 26	40 ± 23	45 ± 28	45 ± 22	40 ± 24	45 ± 28			
General health	L.m	84 ± 14	88 ± 10	85 ± 13	89 ± 10	90 ± 11	85 ± 24	85 ± 13	91 ± 9	87 ± 12	92 ± 9	86 ± 14	90 ± 11	86 ± 14	91 ± 11	0.44	0.03	0.77
	Sp	84 ± 11	87 ± 12	87 ± 12	90 ± 10	90 ± 8	92 ± 9	92 ± 9	91 ± 7	92 ± 8	93 ± 7	88 ± 19	93 ± 8	89 ± 11	89 ± 11			
Bloating	L.m	14 ± 16	15 ± 18	12 ± 18	13 ± 18	11 ± 16	8 ± 15	8 ± 9	5 ± 7	7 ± 15	5 ± 8	9 ± 11	5 ± 12	6 ± 8	6 ± 7	0.09	0.34	0.37
	Sp	11 ± 13	5 ± 5	7 ± 11	4 ± 7	7 ± 9	3 ± 4	2 ± 4	3 ± 3	4 ± 5	8 ± 13	3 ± 4	3 ± 4	9 ± 15	3 ± 4			
Belching	L.m	12 ± 24	9 ± 21	10 ± 19	4 ± 8	6 ± 9	12 ± 22	8 ± 12	5 ± 7	3 ± 3	3 ± 6	6 ± 9	3 ± 4	3 ± 5	3 ± 4	0.44	0.58	0.31
	Sp	7 ± 9	2 ± 3	1 ± 2	3 ± 7	4 ± 10	2 ± 5	2 ± 4	6 ± 9	7 ± 17	7 ± 18	5 ± 6	4 ± 8	7 ± 15	3 ± 4			
Flatulence*	L.m	12 ± 14	15 ± 20	15 ± 16	16 ± 20	24 ± 30	19 ± 17	11 ± 9	20 ± 24	10 ± 12	15 ± 18	13 ± 13	14 ± 13	13 ± 20	10 ± 9	0.02	0.65	0.55
	Sp	18 ± 14	10 ± 17	12 ± 16	8 ± 15	8 ± 13	4 ± 5	4 ± 6	8 ± 10	11 ± 16	10 ± 11	9 ± 10	9 ± 11	10 ± 15	7 ± 7			
Diarrhoea	L.m	1 ± 2	2 ± 3	7 ± 15	13 ± 26	6 ± 11	3 ± 5	3 ± 4	2 ± 4	2 ± 2	2 ± 2	3 ± 4	2 ± 2	2 ± 2	3 ± 3	0.43	0.67	0.13
	Sp	2 ± 2	3 ± 5	2 ± 2	1 ± 1	1 ± 1	2 ± 2	3 ± 4	3 ± 7	3 ± 6	3 ± 6	2 ± 3	4 ± 5	4 ± 7	8 ± 19			
Nausea	L.m	8 ± 18	4 ± 5	5 ± 8	2 ± 3	4 ± 6	7 ± 17	7 ± 12	2 ± 4	1 ± 2	4 ± 7	4 ± 4	2 ± 4	3 ± 3	3 ± 3	0.46	0.77	0.83
	Sp	2 ± 2	1 ± 1	4 ± 6	7 ± 14	3 ± 8	3 ± 4	7 ± 18	3 ± 5	2 ± 2	1 ± 2	2 ± 2	4 ± 6	2 ± 3	4 ± 5			
Constipation	L.m	3 ± 6	10 ± 20	3 ± 4	3 ± 6	11 ± 17	9 ± 16	7 ± 12	5 ± 8	7 ± 16	4 ± 6	7 ± 13	10 ± 15	2 ± 3	5 ± 6	0.04	0.18	0.60
	Sp	3 ± 2	2 ± 3	2 ± 2	1 ± 1	3 ± 2	3 ± 5	6 ± 14	2 ± 3	2 ± 3	2 ± 2	2 ± 2	6 ± 13	4 ± 7	4 ± 5			
Stool consistency	L.m	3.6 ± 0.7	3.8 ± 1	3.8 ± 0.9	4.3 ± 1.4	4 ± 0.8	3.7 ± 1.1	3.5 ± 0.8	3.8 ± 0.8	3.6 ± 0.5	3.8 ± 0.7	4 ± 1	3.3 ± 1.1	3.1 ± 1.1	3.5 ± 1.4	0.69	0.12	0.38
	Sp	3.6 ± 0.7	3.6 ± 0.8	4.1 ± 0.9	3.5 ± 1.2	3.4 ± 0.9	3.6 ± 0.5	3.1 ± 0.8	3.5 ± 1.3	3.8 ± 0.9	3.8 ± 1.4	4.3 ± 1.3	3.4 ± 0.7	4.1 ± 1.2	3.1 ± 0.6			

Mean ± SD on a visual analogue scale (VAS) ranging from 0 to 100. *n* = 12 for each intervention. Days 1–11 represent the intervention days, days 12–14 is the wash out period after the intervention. *P*-values calculated via mixed model ANOVA with intervention (I), time (T) and the interaction intervention-time (I*T)

### Effect on Health Parameters

Table 2 shows the change in fasting plasma parameters and blood pressure at day 12 as compared to day 1. None of the analyzed blood and urine parameters showed a significant change during this period nor did parameters of blood pressure and heart rate.

**Table 2:** Change in fasting plasma parameters, urinary oxalic acid and blood pressure upon 11 days duckweed (*n* = 12) or spinach (*n* = 12) intervention. Data are expressed as Mean ± SD

	Duckweed		Spinach		Group
	Before	Change after 11 days	Before	Change after 11 days	comparison^1^ P Value
Blood					
- Hb (mmol/L)	8.6 ± 0.5	0.0 ± 0.5	8.4 ± 1.1	−0.2 ± 0.4	0.238
- Leukocytes (#/nL)	5.58 ± 1.34	−0.48 ± 1.28	6.20 ± 1.09	−0.83 ± 0.85	0.436
Kidney function					
- Creatinine (umol/L)	79 ± 12	1 ± 5	75 ± 12	−1 ± 7	0.374
- EGFR CKD-EPI (ml/min/1.73 m2)	81 ± 26	6 ± 27	81 ± 26	9 ± 26	0.843
- Urinary oxalic acid (mmol/L)	0.25 ± 0.10	0.13 ± 0.17	0.25 ± 0.11	0.08 ± 0.14	0.459
Liver function					
- ALAT (IU/L)	24 ± 5	−2 ± 4	24 ± 6	−4 ± 4	0.134
- Gamma GT (IU/L)	16 ± 5	−3 ± 2	15 ± 7	−5 ± 3	0.132
Inflammation					
- CRP (mg/L)	3 ± 1	0 ± 1	3 ± 1	1 ± 5	0.430
Iron metabolism					
- Iron (umol/L)	18 ± 4	3 ± 8	17 ± 11	1 ± 8	0.535
- Transferrin (g/L)	2.7 ± 0.5	−0.1 ± 0.2	3.0 ± 0.6	−0.1 ± 0.3	0.938
- Transferrin satur. (%)	27 ± 9	6 ± 15	24 ± 18	1 ± 13	0.443
- Ferritin (μg/L)	67 ± 43	−8 ± 19	67 ± 74	−3 ± 14	0.502
Glucose metabolism					
- Glucose (mmol/L)	4.7 ± 0.4	−0.1 ± 0.4	4.6 ± 0.2	−0.1 ± 0.2	0.899
Cardiovascular					
- SBP (mmHg)	114 ± 11	0 ± 7	107 ± 8	1 ± 6	0.800
- DBP (mmHg)	68 ± 11	−3 ± 7	60 ± 8	1 ± 6	0.136
- HR (bpm)	63 ± 8	0 ± 9	67 ± 11	−1 ± 6	0.640

^1^Calculated by unpaired T-test on change after 11 days (D12-D1)

Oxalate intake has been proposed to link with kidney stone formation. Diets low in oxalate and normal or high in calcium (800–1,200 mg/day for adults) can reduce the urinary excretion of oxalate. Vice versa, a diet rich in oxalates and/or a diet low in calcium increases urinary oxalate [18]. Spinach is known for its relatively high content of oxalate and can contribute to prevalence of high urinary oxalate excretion among idiopathic calcium oxalate stone formers [20]. The oxalic acid and calcium oxalate in the *L .minor* plant material was 70–110 and 100–160 mg/100 g fresh weight, respectively. For steamed or boiled spinach it was reported to contain between 460 and 800 mg/100 g fresh weight, respectively [21]. Our study resulted in an increase in urinary oxalic acid for both intervention arms, but not significantly different between the spinach and the *L. minor* group. Based on this result, it might be concluded that for people with high risk in recurrent kidney stones high intake of *L. minor* should be avoided together with spinach, rhubarb, and other products [22].

Spinach is known for its excellent iron content but it is also shown that the body can only absorb 1.4–7% of the iron present in vegetables [23]. *L. minor* plant material even showed a higher concentration of iron. However, both interventions did not led to a significant change of iron, iron saturated transferrin or ferritin.

The group of Shai performed controlled trials with duckweed material [14, 15, 24]; a single intake study of Wolffia at a level of 30 g protein (410 g fresh weight) in a meal [14], an intervention with 75 g fresh weight Wolffia for three consecutive days [15] and a human intervention trial in which 100 subjects consumed *W. globosa* [24]. In none of the studies adverse events were reported and therefore in line with our results.

### Sensory Evaluation

On average, the basic meals were scored similarly of which the risotto was least appreciated (Supplementary fig. S1). Only for pasta a statistical (*p* < 0.05) difference was found for the overall liking, mouthfeel and aftertaste on the first test day between *L. minor* and spinach, and a statistical difference for the aftertaste of the quiche consumed on day 7 (supplementary fig. S1). From the sensory scores it is clear that *L. minor*- and spinach-based products were almost equally appreciated. Subjects in the Netherlands consume spinach regularly and are therefore used to the typical flavor and texture of this vegetable. The subjects have been told that they would be in either the *L. minor* or the spinach group and we did not intend to mask the flavor and texture. The significant difference that was found on test day 1 could be caused by some degree of food neophobia, the reluctance to eat new or unfamiliar foods [25]. The subjects were volunteers, knowing that they had to consume a new food product, and people with severe food neophobia will likely not subscribe for such study. However, we did not test for this with a validated questionnaire [25] which could be advised to include in similar future studies.

Another observation from the sensory scores is that the evaluations in the second week were similar compared to the first week. When consuming the same product 11 days in a row one might get bored by the taste, but we do not observe this in our data. The taste of *L. minor* is not very strong and may be something we easily get used to. When the food is approved for human consumption a large scale analysis should confirm this further.

## Conclusion

The results from this study show that healthy adults can consume *L. minor* as vegetable up to 170 g for 11 days in a row without any adverse effects, just as could be concluded for the control intervention based on spinach. Therefore, we also expect that consumption of *L. minor* as vegetable will not have adverse effects in a normal dietary pattern where it likely will be consumed less often or in a lower quantity. Together with the knowledge that it is regularly consumed in Asian countries, allowed by the FDA and no adverse events have been reported in other controlled trials, we would consider this product safe for human consumption. We would plead for an introduction of this new food in Europe when the Novel Food application has been approved. Although in depth life cycle analysis of a final commercial production system still has to confirm this, we expect that *L. minor* as vegetable is a very sustainable crop.

The currently used human trial design can be a blue print to study safety and tolerance of other novel foods for which market entrance is foreseen and animal-based studies are undesirable, as this will also increase the sustainability and acceptance of our food system.

## Supplementary Information

ESM 1 (DOCX 161 kb)

## Data Availability

The datasets generated during and/or analyzed during the current study are available from the corresponding author on reasonable request.

## Abbreviations

AE: Adverse events
ALAT: Alanine-aminotransferase
BMI: - Body mass index
CRP: C-reactive protein
dBp: Diastolic blood pressure
eGFR: Estimated glomerular filtration rate
FW: Fresh weight
GGT: Gamma-glutamyl transferase
HR: Heart rate
ICD: International Classification of diseases
MD: Medical doctor
sBp: Systolic blood pressure
VAS: Visual analogue scale
