# Supplementary material for: Daily Intake of Lemna minor or Spinach as Vegetable Does Not Show Significant Difference on Health Parameters and Taste Preference
Source: Plant Foods Hum Nutr. 2022 Feb 12;77(1):121–7. doi: 10.1007/s11130-022-00952-9 (PMC8993727; doi:10.1007/s11130-022-00952-9)
Supplement: Supplementary file 1 — (DOCX 161 kb) [file 11130_2022_952_MOESM1_ESM.docx]

**Supplementary data**

**Supplementary Table S1**. General characteristics of the subject in both arm of the parallel trial.

|  | *L.minor* (*n*=12) | Spinach  (*n*=12) | p values |
| --- | --- | --- | --- |
| Age (y) | 28.6 ± 8.7 | 28.3 ± 8.6 | 0.94 |
| BMI | 22.5 ± 2.0 | 22.4 ± 1.3 | 0.85 |
| Females/Males | 7/5 | 7/5 | N/A |
| Energy intake (kcal) | 1969 ± 449 | 2035 ± 824 | 0.80 |
| Fibre intake (g/d) | 25 ± 6 | 29 ± 9 | 0.55 |
| Plant protein (g/d) | 37 ± 12 | 41 ± 15 | 0.48 |

Supplementary Table S2. General overview of nutrient composition of *L.minor* and spinach.

|  |  | *L.minor*  (100g FW) | Spinach ^#^  (100g FW) |
| --- | --- | --- | --- |
| Macronutrient | Energy (kcal) | 11 | 24 |
|  | Protein (g) | 2 | 2.3 |
|  | Total lipid (g) | 0.15 | 0.4 |
|  | Fatty acids total saturated (g) | 0.06 | 0 |
|  | Fatty acids, total trans (g) | 0 | 0 |
|  | Cholesterol (mg) | n.s | 0 |
|  | Carbohydrates (by difference) (g) | n.s | 1.7 |
|  | Sugars total (g) | 0 | 0.9 |
|  | Fibre, total dietary (g) | 0.85 | 2.4 |
| Minerals | Calcium (mg) | 99 | 162 |
|  | Iron (mg) | 7.6 | 1.2 |
|  | Sodium (mg) | 4 | 25 |
| Vitamins | Vitamin C (mg) | 9 | 8 |
|  | Folate (µg) | n.s. | 63 |
|  | Vitamin A (RAE) | n.s. | 321 |
|  | Vitamin E (mg) | 3.5 | 1.7 |

^#^ based on NEVO code 146. n.s. (not specified)

**Supplementary Tabel S3**. Ingredients per type of dish.

| DISH | Ingredients per portion |
| --- | --- |
| Pasta | 170 g *L. minor* or spinach, 146 g sauce (41 g mushrooms, 36.7 g Boursin, 35 g onion, 31 g lean bacon, 2.3 g vegetable broth powder), 134 g penne |
| Curry | 170 g *L. minor* or spinach, 146 g sauce (57 g chicken, 39 g coconut milk, 25 g mild green boemboe herbal mixture, 21 g pineapple, 4 g olive oil), 134 g rice, |
| Mashed potato | 170 g *L. minor* or spinach, 280 g potato mixture (155 g potato, 48 g sun dried tomato in oil, 38 g lean bacon, 15 g pine seeds, 15 g liquid baking margarine, 9 g semi-skimmed milk) |
| Risotto | 170 g *L. minor* or spinach, 280 g risotto mixture (95.7 g water, 40 g mushrooms, 39 g risotto rice, 39 g ricotta, 36 g celery, 18 g onions, 9 g olive oil, 3 g vegetable broth powder, 0.3 g Italian herbal mixture) |
| Soup | 170 g *L. minor* or spinach, 266 g soup (169 g water, 41 g onion, 27 g red pesto, 19 g crème fraiche, 6.7 g vegetable broth powder, 3.3 g margarine), 14 g Grand Padano cheese |
| Lasagne | 170 g *L. minor* or spinach, 230 g sauce (95 g tomato sauce, 45 g water, 42.7 g beef mince, 18 g onion, 17 g mozzarella, 10 g olive oil, 2 g vegetable broth powder, 0.3 g Italian herb mixture), 90 g lasagne sheets, 14 g Grand Padano cheese |
| Quiche | 170 g *L.minor* or spinach, 145 g sauce (70 g egg, 34 g zucchini, 23 g cashew, 15 g cooking cream, 1.5 g olive oil, 1.5 g vegetable broth powder, 0.1 g thyme), 113 g puff pastry, 50 g Brie 60+ |


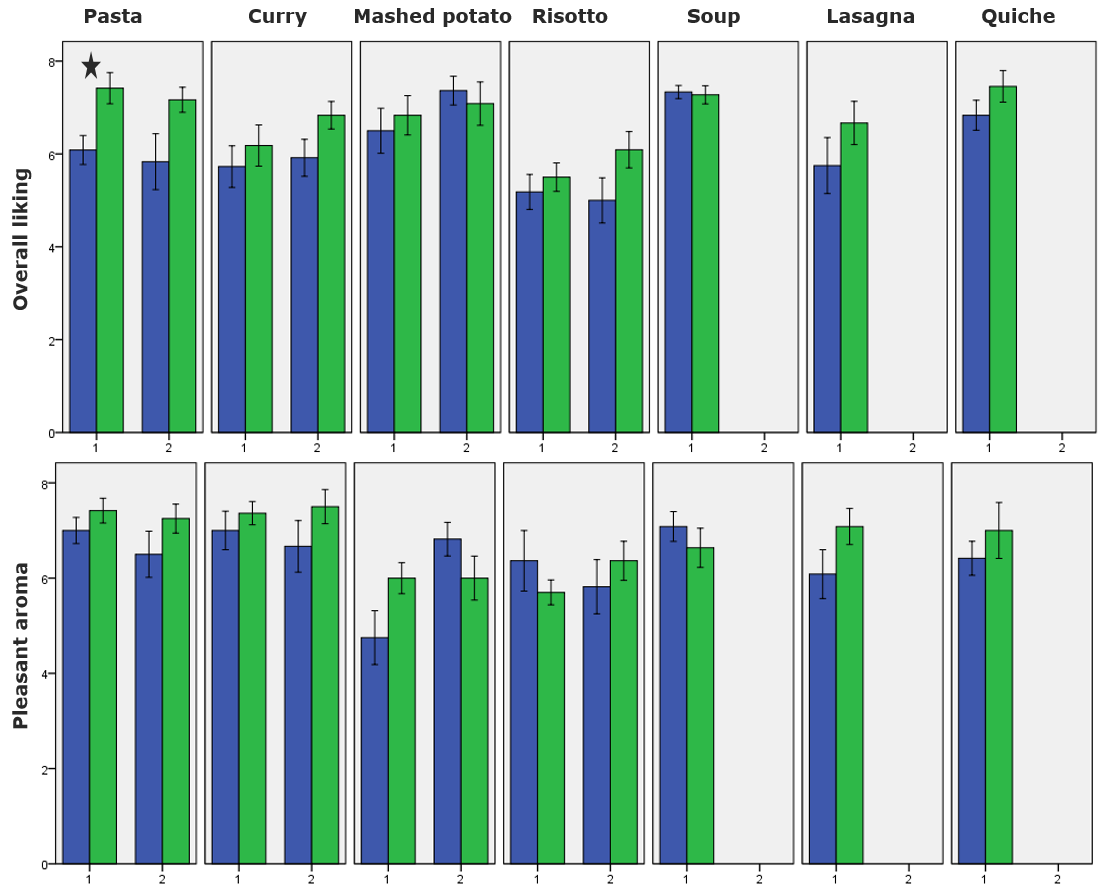


**A**

**B**


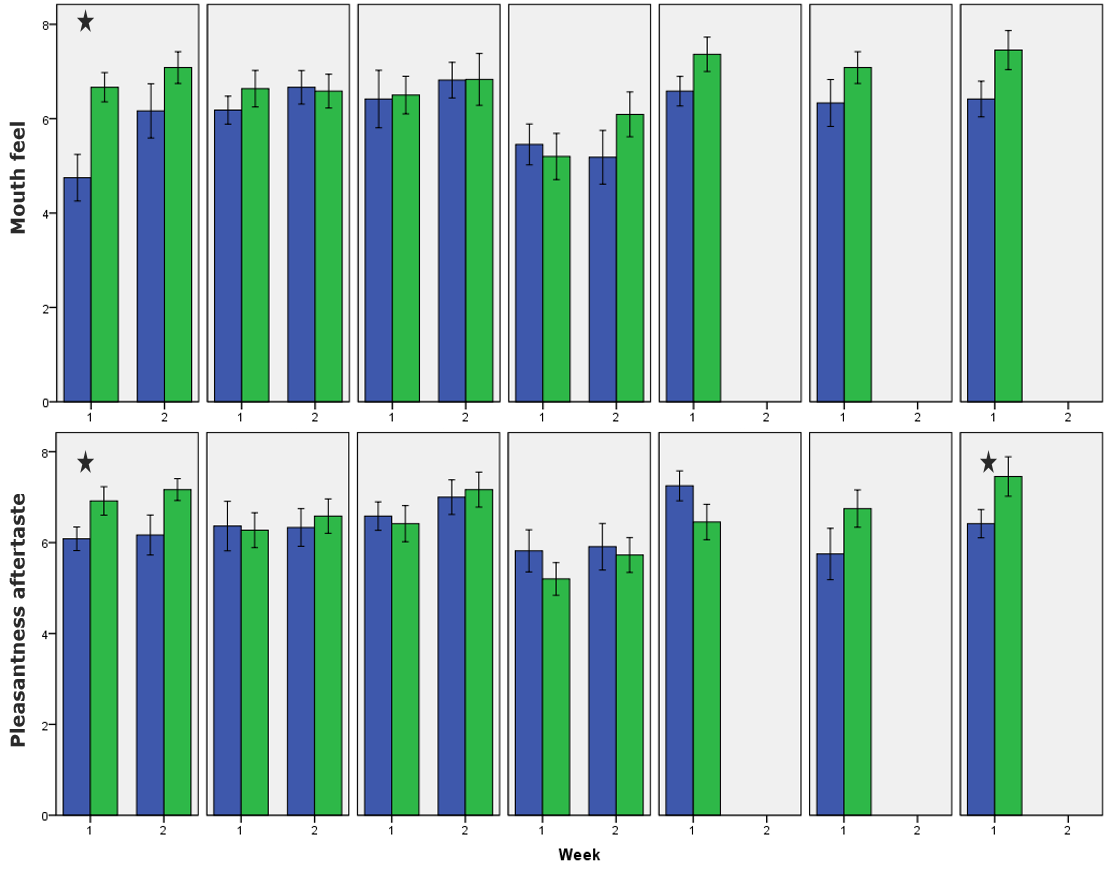


**C**

**D**

**Supplementary Fig S1.** Sensory analysis of the products per product day. Attributes were evaluated on a 10 point scale. Panel A indicate the overall liking of the products, panel B the aroma, panel C the mouth feel and panel D the pleasantness of the aftertaste. Blue bars represent the *L .minor*-based product, the green bars represent the spinach-based products. * indicate statistical difference between *L. minor* and spinach (p<0.05).
